# Supplementary material for: Patterns of Intron Gain and Loss in Fungi
Source: PLoS Biol. 2004 Nov 30;2(12):e422. doi: 10.1371/journal.pbio.0020422 (PMC532390; doi:10.1371/journal.pbio.0020422)
Supplement: Table S1 — Also available at http://genes.mit.edu/NielsenEtAl/. (4.3 MB ZIP). [file pbio.0020422.st001.zip › NielsenEtAl/html/751.html]

AN6614.1.NCU03818.1.MG04066.1.FG05149.1


```
 CLUSTAL W (1.82) Multiple Sequence Alignments - Introns Inserted


Sequence 1: MG04066.1	1376 aa
Sequence 2: FG05149.1	1304 aa
Sequence 3: NCU03818.1	1331 aa
Sequence 4: AN6614.1	1265 aa
Alignment Length: 1406 aa
Number Identitical Residues: 663 aa
Alignment Score (without introns) 30275


MG04066.1 	MPNQQASHHHPSKPPDS-PVDDSDSDLEIDLQELDPQSNSSQRPLRGSAESYAREAAEHR
NCU03818.1	MPSSDT--YHLSKPPDSHVVDESDSDLEIDLEELDPQATDPNNHHQSLGQHRSRNSSEQR
FG05149.1 	MPPSQQ--YRPSDPPDS-PTNDSDSDLDLDIQELDPISTTPAP--RATKEHPPTEPETSR
AN6614.1  	MPSSSE--YLRAHDRAS---IESDND-DLDLEELDPSSAFNAS--RLSRDEPVQQRGHSG
          	** ..   :  :.   *    :**.* ::*::**** :       :   :    :     

MG04066.1 	SSP---RIPLRNLRMGGLRRSRDRGQ-YGELGHRRDGSSDQSAGLLTPGDRDASSSRFED
NCU03818.1	PPPPTTSIALRNLRMGSLRRGAKRNG-YGELGRSRDGNDDDDADALLRHDRHSVGSATHD
FG05149.1 	-------IALRNLRMGGLRRAGKRNRGYGDLGRDRDGNDEHSEALL--GDRNSSTPQWSE
AN6614.1  	P-----GIALRNLRVGVGNRRLWKRSMSGSQGEDTAGLLEDREGERGLRPSHASSRPYTD
          	.      *.*****:*  .*   :    *. *.   *  :.          .:      :

MG04066.1 	SDR---HGDDDAPLLP-DSEQGRRRPGGHRRLKSITDALRLPSFMSSSSNVADQRQPLPE
NCU03818.1	EDAGLLSEHGGGGRIRTSSGRRRRSFAGDG---LRNISMKLPGFMSGD-------QSNHN
FG05149.1 	GSG---YNEDDQPLLGGQPSQGRRSINSDR----VSSRLRLPSFMSSS------KKSDTN
AN6614.1  	DDAP---LLDRRGSVRSLADGGRMPQRRSR-----LGWLKSSTLLGGL--------HGST
          	 .       .    :   .   *               :: . ::..             

MG04066.1 	EDEGQEDHDPSGSRLVAVGSAQSARFPPNAVSNAKYTAWSFLPITLYNEFSFFFNMYFLL
NCU03818.1	DEQEQEEDDPSSSRLVAVGSSQSTRFPPNIISNAKYTAWSFLPVTLYNEFSFFFNMYFLL
FG05149.1 	DAEDREDDDPSSSRHVAVGSTQPVRFPNNIISNAKYTALTFLPITLYNEFSFFFNMYFLL
AN6614.1  	GGGKNTVDVVKPPRDVLVGQAQRAKYPPNIVSNAKYTPWSFLPRTLYNEFSFFFNIYFLL
          	    .  .  . .* * **.:* .::* * :******. :*** ***********:****

MG04066.1 	VALSQAIPALRIGYISTYIAPLAFVLMITLGKEAHDDIARRRRDSEANSEPYTTIQFDDP
NCU03818.1	VALSQAIPALRIGYLSTYVAPLAFVLFITLAKEAYDDIERRRRDNEANSEEYTVLQFDDP
FG05149.1 	VALSQAIPALRIGYLLTYIAPLAFVLCITMGKEAFDDIARRRRDTEANSEEYKVIVFQDA
AN6614.1  	VALSQIIPVLRIGYMSTYIAPLAFVVSISLGKEALDDVGRRRRDAEANAEEFSVLALD--
          	***** **.*****: **:******: *::.*** **: ***** ***:* :..: ::  

MG04066.1 	GATVKNGRSRKKLKSAGSRKAQLRLSE---RDRLSDIQEEEELAEGDGAGKGASSHIREV
NCU03818.1	GASLGINRPRRKMKSSHTRTGSKRLGIEQENDRLSDIQEEEEQTEGRGLRELPASYLSEV
FG05149.1 	DLTQTPVRQRKLLKSEVLQKHSRKKSKG-ARENLSDILEEENVEAPP-----PSSRAIEV
AN6614.1  	-------------KSGGSRAFP--------------IAEDVG------------ESVPVI
          	             **   :                 * *:              .    :

MG04066.1 	LKASKDLKVGDVLRLTKGQRVPADVVILQCLASEVTASTPVASK--LAEPELLTLDDAS-
NCU03818.1	SKKSRDLKVGDVLKLTKGHRVPADVIILKCLAHESAANKETEEQEVPAKEEMLLLDHVDD
FG05149.1 	SRKSKDLKVGDVLKLTKGQRVPADVVILKCLSSEVPAPAPIPEE--PAEEETLLAFDGE-
AN6614.1  	IKKSRDLKVGDVLKVRKNQRLPADVVILKSVSNDATVARESQEAG--ASHNVDYATNGS-
          	 : *:********:: *.:*:****:**:.:: : ..     .    *. :     . . 

MG04066.1 	---GNGGASQPRDDGASDGDGSQAGAGGETFIRTDQLDGETDWKLRIASPLSQNLATEEL
NCU03818.1	DDVGEGSSKNAKTSKESNNNGAEGSSSGETFIRTDQLDGETDWKLRLASPLSQNLSTEEL
FG05149.1 	---EPQGKGKQPAEAAPEQE-AESGPGGETFIRTDQLDGETDWKLRLASPLTQNLPTEEF
AN6614.1  	----SSSVPNQQPTISAGADQTDISSASDTFIRTDQLDGETDWKLRLPSVLSQALSLEDF
          	      .  :      .  : :: ....:*****************:.* *:* *. *::

MG04066.1 	VRFRVTAGKPDKKVNEFVGTLELLPS------RQDAMSPGARLPNGDELANTAPLTIDNT
NCU03818.1	VRLRVTAGKPDKRVNEFVGTLELLPS------RQDVMSGAAYNPREGDDVKAAPLSIDNT
FG05149.1 	VRLRVTGGKPDRKVNEFLGTIELVES------RKDALAHHATVGESD-ASHTAALSIDNT
AN6614.1  	TRLKITASGPDKRVNEFVGNIELLPPSGFYDPHVDKSEDRSQDADETEQNSSAPLTIDNT
          	.*:::*.. **::****:*.:**: .:.  ..: *     :      .   :*.*:****

MG04066.1 	AWANTVIASRAVTLAVIVYTGPQTRSALSTSPSRSKTGLLEYEINSLTKILCALTLALSI
NCU03818.1	AWANTVIASNATTLAVIVYTGPQTRSALSTSPSRSKTGLLEYEINSLTKILCFLTLFLSI
FG05149.1 	AWANTVIASQATTLAVIMYTGPQTRSALSTAPSRSKTGLLEYEINSLTKILCALTLALSI
AN6614.1  	AWANTVLASNTITYAAIIYTGSQTRAALSTSPSRSKVGLLEYEINNLTKILCALTLTLSI
          	******:**.: * *.*:***.***:****:*****.********.****** *** ***

MG04066.1 	ILVAFQGFTNTEGNVWYIKIMRFLVLFSTIVPISLRVNLDLGKSVYSWFIQRDPGMPGAV
NCU03818.1	VLVALEGFSTAKGNIWYVKIMRFLVLFSTIVPISLRVNLDLGKSVYSWFIQRDPGIPGAV
FG05149.1 	ILVALEGFGNTKDNVWYVKIMRFLVLFSTIVPISLRVNLDMGKSAYSWFIQRDPGMPGAV
AN6614.1  	ILVALEGFQPTNDKEWYVAIMIYLILFSTIIPMSLRVNLDMAKSVYGRFIERDKDIPDTV
          	:***::**  ::.: **: ** :*:*****:*:*******:.**.*. **:** .:*.:*

MG04066.1 	VRTSMIPEDLGRIEYLLSDKTGTLTQNE~MEMKKIHIGTVSYANEAMDEVSSYIKQGFHV
NCU03818.1	VRTSTIPEDLGRIEYLLSDKTGTLTQNE~MEMKKIHVGTVSYANEAMDEVATYVKQGFNL
FG05149.1 	VRTSTIPEDLGRIEYLLSDKTGTLTQNE~MEMKKIHVGTVSYANEAMDEVSAYVKQGFYV
AN6614.1  	VRTSTIPEDLGRIEYLLSDKTGTLTQNE1MELKKIHVGTVSYANDAMEEVASYVRQSFSG
          	**** *********************** **:****:*******:**:**::*::*.*  

MG04066.1 	QPTLSDTSESIQLVTPSSTFTNAAASGTTRTRREIGARVRDVVLALALCHNVTPTTDE-E
NCU03818.1	SSSAS--SASLALATPSSTYS-AANVGATRTRREIGSRVRDVVLALALCHNVTPTSEEDE
FG05149.1 	PPSTDHVSQN-MLITPSSTYSSSANIGTTRTRREIGTRVRDVVLALALCHNVTPTVDI-E
AN6614.1  	NTLTS---------AAAAFGTQAGLAAAPRTRREIGSRVRDIVLALALCHNVTPTSDE-E
          	 .  .         :.::  :.:.  .:.*******:****:************* :  *

MG04066.1 	DGKTVTSYQASSPDEIAIVKWTEAVGLRLTYRDRQSMTLSFSETGKTVVHVRILDIFPFT
NCU03818.1	NGHTVNSYQASSPDEIAIVKWTESVGLRLAHRDRKNIVLESAETGRPVVKVRILDIFPFT
FG05149.1 	DGKEVTGYQASSPDEIAIVKWTESVGLKLVYRDRKSMVLEYANSKRPVVRVRILDVFPFT
AN6614.1  	DGVKVTNYQASSPDEIAIVRYTEEVGLKVAYRDRRTIVLESTDTGNVVVRARILDIFPFT
          	:*  *..************::** ***::.:***:.:.*. ::: . **:.****:****

MG04066.1 	SEGKRMGVIVQFFERPNE----IGSGETWFYQKGADTVMSSIVAANDWLDEETANMAREG
NCU03818.1	SEGKRMGIIVQFYEKLQPGNPSLGSSEIWFYQKGADTVMSSIVAANDWLDEETANMAREG
FG05149.1 	SEGKRMGIIVHFHEDASVKNPSLSSGEIWFFQKGADTVMSSIVASNDWLDEETANMAREG
AN6614.1  	SDSKRMGIIVEFDKDKDVLN-SPAEEEIWFYQKGADTVMTSIVAANDWLDEETANMAREG
          	*:.****:**.* :  .  . : .. * **:********:****:***************

MG04066.1 	LRTLVVGRKRLSAEQYREFSSRYQEASLAINGRDAGMQKVVSHYLERDLELLGVTGVEDK
NCU03818.1	LRTLVVGRKRLSLKEYQEFSAKHHEASLAISGREANMQAVVSQYLEHNLELLGVTGVEDK
FG05149.1 	LRTLVVGRKKLSYGQYQEFAARYQTASLSISGRDAGMQRVVSHYLENELELIGVTGVEDK
AN6614.1  	LRTLVVGRKRLSPLQYQEFASKYKQASLSLQGRDIGMQKVVNEYLEHDLELLGVTGVEDR
          	*********:**  :*:**::::: ***::.**: .** **..***.:***:*******:

MG04066.1 	LQKDVKPSLELLRNAGIKIWMLTGDKVETARCVAVSSKLVARGQYIYTLAKMKRTDSAQD
NCU03818.1	LQKDVKPSLELLRNAGIKIWMLTGDKVETARCVAVSSKLVARGQYIHTIAKLRRKDGAQD
FG05149.1 	LQKDVKPSLELLRNAGIKIWMLTGDKVETARCVAVSSKLVARGQYIYTVSKLTKKDNAQE
AN6614.1  	LQRDVKPSLELLRNAGVKIWMLTGDKVETARCVAISAKLVARGQYIHTVAKVKDKSAAQE
          	**:*************:*****************:*:*********:*::*:  .. **:

MG04066.1 	HLDFLRNKTDACLLIDGESLALLLQYHRLDFVSVAVRLPTVVACRCSPTQKAEVARLIKD
NCU03818.1	NLDLLRSKTDSCLLIDGESLAMYLTHFRREFISVAVLLPTVVACRCSPTQKAEVAKLIKE
FG05149.1 	HLDFLRSKTDACLLVDGDSLALFLTHFRLEFISIAVQLPTVVACRCSPNQKAEVAKLIRE
AN6614.1  	ALDFLRNKTDCCLLIDGESLALMLNQFRSAFISVAVLLPAVIACRCSPTQKAEVADLIRL
          	 **:**.***.***:**:***: *  .*  *:*:** **:*:******.****** **: 

MG04066.1 	YTRKRVCCIGDGGNDVSMIQAADVGVGIVGKEGRQASLAADFSIEQFHHLTKLLVWHGRN
NCU03818.1	YTKKRVCCIGDGGNDVSMIQAADVGVGIVGKEGRQASLAADFSIEQFHHLTKLLVWHGRN
FG05149.1 	YTKKRVCCIGDGGNDVSMIQAADVGVGIVGKEGRQASLAADFSIEQFCHLVKLLVWHGRN
AN6614.1  	HTKKRVCCIGDGGNDVSMIQAADVGIGIVGKEGRQASLAADFSITQFHHITKLLVWHGRN
          	:*:**********************:****************** ** *:.*********

MG04066.1 	SYKRSAKLAQFVIHRGLIIAVCQTMYSIAMHFEPDGLYK0DWLLVGYATMYTAFPVLSLV
NCU03818.1	SYKRSAKLAQFVIHRGLIIAVCQTMYSIALKFEPEGLYK0DWLMVGYATVYTAFPVLSLV
FG05149.1 	SYKRSAKLAQFVIHRGLIIAVCQTMYSIAIKFEPEGLYK0DWLLVGYATCYTAAPVLSLV
AN6614.1  	SYKRSAKLAQFIMHRGLIISACQTMYSIASHFDPKGLFI~NWLMVGYATVYTNAPVFSLV
          	***********::******:.******** :*:*.**:  :**:***** **  **:***

MG04066.1 	LDKDVDENLANLYPELYKELTSGRSLSYRTFFVWVLVSIYQGGIIQGLSQLLTE-VDGPK
NCU03818.1	LDKDVDEDLANLYPELYKELTSGSSLSYRTFFVWVFVSIYQGCLIQGLSQVLTG-IDGPR
FG05149.1 	LDKDVDEDLANLYPELYKELTTGRSLSYRTFFVWVLVSIYQGGMIQGLSQILTE-VDGPK
AN6614.1  	LDRDVDEQLANLYPELYKELKTGRSLSYRSFFTWVLVSVYQGAVIQGLSQILLDTISGKR
          	**:****:************.:* *****:**.**:**:*** :******:*  ::.* :

MG04066.1 	MVAVSFTVLVLNELLMVAIEITTWHPVMIVSIVGTFLVYMGSIPFLSGYFDLKFMITL2K
NCU03818.1	MLAVSYTVLVLNELLMVAIEITTWHWVMVVSIVGTFLMYIVSIPFLGDYFDLKFVLTL2G
FG05149.1 	MVAVSYTVLVLNELLMVAIEITTWHPIMIISIIGTFLLYIGSIPFLGGYFDLEFLITW2G
AN6614.1  	LISVSFTALVMNELLMVAIAVTTWHPVMIFCLLGTALVYAASVPFLGEYFDLKYVITL~D
          	:::**:*.**:******** :**** :*:..::** *:*  *:***. ****::::*   

MG04066.1 	VQE1LMVDFLRLQPQQQVTDKRRGGRMVTDLESKNPIKDELVRRLKSPHV0DLNPSHNPL
NCU03818.1	---~FLWRVAAIAAISLIP----------------TYAAKVIRRTMKP--~---PSYRKV
FG05149.1 	---~FVWRVLAIGAISLVP----------------PYAGKLIRRTMKP--~---PSYRKV
AN6614.1  	---~WIWRVVAVTAVSVIP----------------VWAAKLIKQSWNP--~---PSYRKV
          	     :  .  : . . :.                    :::::  .*      **:. :

MG04066.1 	KSTRRLSVDAVNAAVEQQPGYDIFVSIVVPA
NCU03818.1	QGI----------------------------
FG05149.1 	QNR----------------------------
AN6614.1  	RG-----------------------------
          	:.
```
